# Severe dysplasminogenemia due to homozygous *PLG* Ala620Thr variant in a Korean woman without a history of venous thromboembolism: A case report and literature review

**DOI:** 10.1097/MD.0000000000029013

**Published:** 2022-03-04

**Authors:** Beomki Lee, Suekyeung Kim, Jae Joon Lee, Seon-Hee Heo, Suryeun Chung, Shin Yi Jang, Sun-Hee Kim, Duk-Kyung Kim, Hee-Jin Kim

**Affiliations:** a Department of Laboratory Medicine and Genetics, Samsung Medical Center, Sungkyunkwan University School of Medicine, Seoul, Korea; b Department of Surgery, Yonsei University College of Medicine, Seoul, Korea; c Department of Thoracic and Cardiovascular Surgery, Samsung Medical Center, Sungkyunkwan University School of Medicine, Seoul, Korea; d Division of Cardiology, Department of Medicine, Heart Vascular Stroke Institute, Samsung Medical Center, Sungkyunkwan University School of Medicine, Seoul, Korea.

**Keywords:** Ala620Thr, dysplasminogenemia, plasminogen, *PLG*, thromboembolism

## Abstract

**Rationale:**

Plasminogen plays an important role in fibrinolysis and is encoded by the *PLG* gene. The missense variant *PLG* Ala620Thr is the major cause of dysplasminogenemia in East Asian countries, including Korea. Although dysplasminogenemia was first reported in a Japanese patient with recurrent venous thromboembolism (VTE), subsequent studies have not demonstrated any clear association between the *PLG* Ala620Thr variant and the risk of VTE. To the best of our knowledge, this is the first report of a homozygous *PLG* Ala620Thr variant case from Korea.

**Patient concerns:**

Here, we report a Korean family with *PLG* Ala620Thr mutation. The proband was a 34-year-old man who presented with multiple thrombotic arterial embolism and cardiac myxoma.

**Interventions:**

Laboratory workup, including coagulation profile and *PLG* gene sequencing, was carried out for the affected family.

**Diagnosis and Outcome:**

The proband carried a heterozygous *PLG* Ala620Thr variant with decreased plasminogen activity of 65%. His 53-year-old mother, who had no reported history of VTE, was homozygous for the *PLG* Ala620Thr variant with decreased plasminogen activity of just 25%. Decreased plasminogen activity indicates dysplasminogenemia.

**Lessons:**

We believe that this clinically silent homozygous case supports the previous findings that isolated *PLG* Ala620Thr variant does not confer a significant risk of VTE.

## 1 Introduction

Plasminogen, encoded by the *PLG* gene on chromosome band 6q26, is the precursor of plasmin, a serine protease that plays an important role in fibrinolysis. Plasminogen deficiency is classified into 2 categories: hypoplasminogenemia (type I) and dysplasminogenemia (type II). Dysplasminogenemia (type II plasminogen deficiency) is a rare genetic condition in which plasminogen activity is reduced, despite a normal level of plasminogen antigen, whereas hypoplasminogenemia (type I plasminogen deficiency) demonstrates decrease in both plasminogen antigen level and activity.^[1]^ There are several known causes for dysplasminogenemia, but one of the most common causes is a genetic variant of the *PLG* gene, Ala620Thr.^[1,2]^ This missense variant, which is caused by a single nucleotide change, NM_000301.3(*PLG*):c.1858G > A, leads to dysplasminogenemia whose association with thrombosis is still controversial.

In the previous studies, several presumed homozygous cases of the *PLG* Ala620Thr variant have been identified based on the reduced level and activity of plasminogen (Table 1). In one such study, 10 homozygotes were identified, however, whether these individuals developed thrombosis was not addressed.^[2]^ Two previous reports of presumed homozygotes with reduced plasminogen activity identified a 40-year-old man with multiple events of thrombophlebitis after contusion,^[3]^ and a 5-year-old boy without any previous thrombotic event.^[4,5]^ Another study from Japan reported 2 and 19 suspected homozygote cases with unusually low plasminogen activity among healthy populations and patients with comorbidity, respectively.^[6]^ However, there has been only 1 case that demonstrated that the genetically proven homozygous Ala620Thr variant does not associate with venous thromboembolism.^[7,8]^ Therefore, to validate the previous finding of dysplasminogenemia nonpredisposition to thrombosis, we hereby report this incidentally found case of a homozygous variant, who did not have any previous history of thrombosis.

**Table 1:** Reported cases with suspected or genetically confirmed homozygous *PLG* Ala620Thr variant.

No.	References	Subjects	Venous thromboembolism	Plasminogen activity	*PLG* sequencing	Age at diagnosis
1	Aoki et al^[4]^ Sakata et al^[5]^	1	None	5.7%	NA	5 yr
2	Kazama et al^[3]^	1	Recurrent thrombophlebitis	18.7%	NA	40 yr
3	Shinmyozu et al^[7]^ Ichinose et al^[8]^	1	None	8.1%	c.1858G > A (Hom)	NA
4	Tsutsumi et al^[2]^	10	NA	NA	c.1858G > A (Hom)	NA
5	Okamoto et al^[6]^	21	None^∗^	1.5%–26.2%	NA	6–85 yr
6	Current case	1	None	25%	c.1858G > A (Hom)	53 yr

∗ While none had experienced deep vein thrombosis, three had stroke, four had myocardial infarction, and four had angina pectoris. Hom = homozygous, NA = not available.

## 2 Case report

**Figure 1:**
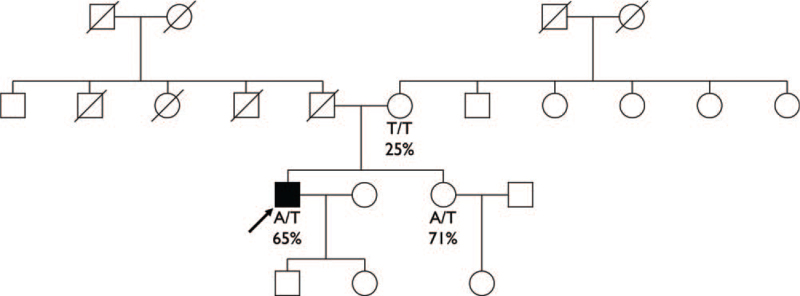
The pedigree of the proband family. The arrow indicates the proband and the filled symbol indicates subject with thrombosis. Below each symbol, the combination of amino acids and the percentage of the plasminogen activity are presented. A = alanine, T = threonine.

A 34-year-old male proband visited the emergency room of another hospital due to sudden dizziness and lower limb weakness. Since contrast-enhanced computed tomography showed renal infarction, splenic infarction, and occlusion of both common femoral arteries, the patient was referred to our institute for emergency surgery. After emergent thromboembolectomy of the bilateral superficial femoral arteries, left common femoral artery, and fasciotomy of the left lower leg, the patient was admitted and underwent additional operations, including removal of cardiac myxoma in the left atrium. During admission, further analysis for the proband's underlying cause of thromboembolism was performed. To measure the coagulation profile, an STA-R analyzer (Diagnostica Stago, Asnieres, France) was used according to the manual instructions. The proband demonstrated decreased plasminogen activity at 65% (reference range 75%–112%) and was otherwise not remarkable. However, in a subsequent familial study, plasminogen activity decreased to 25% in the proband's 53-year-old mother and 71% in the proband's 33-year-old younger sister, which indicated plasminogen deficiency. Since the proband's mother showed an even greater decrease in plasminogen activity compared to other family members, we suspected her to be the case of either homozygous or compound heterozygous *PLG* genetic variants (Fig. 1). Therefore, to confirm the underlying genetic cause of reduced plasminogen activity, we sequenced the *PLG* gene from the proband and his family members.

**Figure 2:**
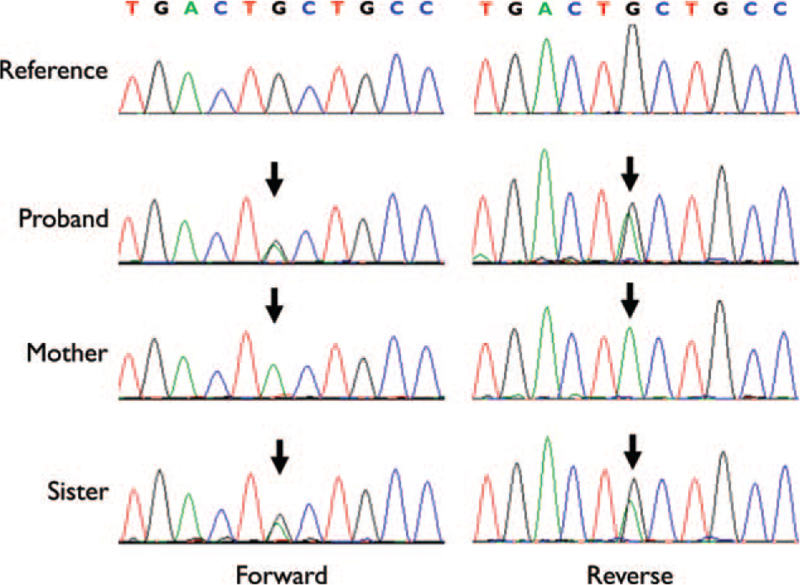
Sequencing analysis of the *PLG* gene. The proband and his sister were heterozygous whereas his mother was homozygous for c.1858G > A (p. Ala620Thr).

DNA was extracted from whole blood using a Roche MagNA Pure 96 DNA isolation kit (Roche Applied Science, Manheim, Germany). All 19 exons of the *PLG* gene were evaluated by polymerase chain reaction (PCR) and full sequencing using an ABI Prism 3730XL DNA sequencer (Applied Biosystems, Foster City, CA, USA). The forward and reverse PCR primers for exons 1 to 19 were designed by the authors and are available upon request. The sequencing results revealed that the proband carries heterozygous *PLG* Ala620Thr. The proband's mother and younger sister had homozygote and heterozygote *PLG* Ala620Thr variants, respectively, but both did not have any history of thrombotic events. Unfortunately, the proband's father was deceased and could not be assessed in this study (Fig. 2).

The proband recovered after emergent thromboembolectomy and cardiac myxoma removal and was treated with warfarin. However, 10 months after the first thrombotic incidence, the proband presented with a newly appearing mass in the right atrium, suggestive of reoccurrence of cardiac myxoma, which was also surgically removed. Nonetheless, the proband's mother carrying homozygous Ala620Thr variant did not have any thrombotic events to present at all.

## 3 Discussion

Plasminogen gets converted to plasmin by peptide bond cleavage at Arg561-Val562 by tissue-type plasminogen activator or urokinase-type plasminogen activator, which plays a crucial role in fibrinolysis by preventing fibrin clot formation.^[1]^ However, dysplasminogenemia predisposition to thrombosis has been debated, since there is only 1 genetically proven homozygous Ala620Thr variant without thrombosis,^[7,8]^ to date. Although dysplasminogenemia was first identified in a patient with recurrent thrombosis,^[4]^ there have been several reports suggesting that dysplasminogenemia itself may not be a risk factor for thrombosis.^[6,9,10]^ Furthermore, in the homozygous *PLG* Ala620Thr mouse model, the risk of thrombotic diseases was not increased despite decreased plasmin activity.^[11]^

In this report, during a familial study of a thrombosis patient diagnosed with dysplasminogenemia due to *PLG* Ala620Thr heterozygosity, a homozygous case was identified unexpectedly. The 53-year-old proband's mother, with *PLG* Ala620Thr homozygosity, did not have any previous history of thrombosis. The 33-year-old female proband's sister, with heterozygous Ala620Thr, also did not have any previous history of thrombosis. These findings support the previous findings that dysplasminogenemia is not a risk factor for thrombosis. However, there may be other coagulative defects leading to thrombosis with or without a synergistic effect with dysplasminogenemia, which is why some patients with heterozygous *PLG* Ala620Thr variant present with thrombosis, like the proband in our report. These mechanisms have yet to be elucidated, and further research is warranted.

The *PLG* Ala620Thr variant is more prevalent in East Asian countries. In the Korean population, the estimated allele frequency is about 1.6%,^[12]^ whereas Korean Reference Genome Database suggested it to be 2.3312%. Applying the Hardy-Weinberg principle, the prevalence of Ala620Thr homozygotes was predicted to be 0.0256% to 0.0543%. Hence, with a Korean population of 52 million, approximately 13,000–28,000 individuals are predicted to carry a homozygous Ala620Thr variant. Despite this large number of estimated homozygote individuals, the number of genetically confirmed cases of dysplasminogenemia in thrombosis patients remains limited, suggesting that the homozygous *PLG* Ala620Thr variant is not related to an increased risk of thrombosis. Given the function of plasmin in clot degradation, dysplasminogenemia seems to be a plausible risk factor for thrombosis, however, the finding of *PLG* Ala620Thr homozygous case with no thrombosis in this report further validates that dysplasminogenemia is not a predisposing factor for thrombosis. It is speculated that decreased plasmin activity may be sufficient for preventing thrombosis in vivo.^[11]^

Although there was only 1 case of genetically proven Ala620Thr homozygote without thrombosis,^[7,8]^ a population-based study from Japan identified individuals with markedly decreased plasminogen activity with some of them suspected to be unrevealed homozygotes.^[6]^ It has been shown that the extent of decrease in the anticoagulant activity of a certain protein could vary depending on the type of mutation and zygosity.^[13]^ According to this study, nonsense mutations showed a greater decrease in anticoagulative activity compared to missense mutations.^[13]^ In addition, the extent of decrease in anticoagulative activity in double missense mutations was comparable to that of nonsense mutations.^[13]^ Similarly, in suspected dysplasminogenemia homozygotes from Japanese study,^[6]^ the possibility of these subjects carrying compound heterozygote variants cannot be ruled out. Since markedly reduced protein activity could result from both homozygous and compound heterozygous mutations, the evaluation of the underlying genetic background is important in elucidating the genotype-phenotype correlation and disease association. Therefore, the current case is clinically significant, as it is only the second report of genetically proven Ala620Thr homozygote without any thrombotic events. This line of evidence further validates classifying this variant as a variant of uncertain significance rather than a pathogenic or likely pathogenic variant according to the ACMG/AMP variant classification system.

In summary, this case is clinically significant since it is the second genetically proven case of homozygous Ala620Thr with no definite evidence of thrombosis. To our knowledge, this is the first report of a homozygous Ala620Thr mutation in *PLG* in Korea. Our report supports the previous findings that the isolated *PLG* Ala620Thr variant does not confer a significant risk of thromboembolism and thus contributes substantially to research on dysplasminogenemia.

## Author contributions

**Conceptualization:** Duk-Kyung Kim, Hee-Jin Kim.

**Data curation:** Beomki Lee, Shin Yi Jang, Hee-Jin Kim.

**Formal analysis:** Suekyeung Kim, Hee-Jin Kim.

**Investigation:** Beomki Lee, Jae Joon Lee, Duk-Kyung Kim, Hee-Jin Kim.

**Resources:** Seon-Hee Heo, Suryeun Chung, Shin Yi Jang, Sun-Hee Kim.

**Writing – original draft:** Beomki Lee.

**Writing – review & editing:** Beomki Lee, Duk-Kyung Kim, Hee-Jin Kim.

## References

[1] Schuster V Hugle B Tefs K. Plasminogen deficiency. J Thromb Haemost 2007;5:2315–22. 17900274 10.1111/j.1538-7836.2007.02776.x

[2] Tsutsumi S Saito T Sakata T . Genetic diagnosis of dysplasminogenemia: detection of an Ala601-Thr mutation in 118 out of 125 families and identification of a new Asp676-Asn mutation. Thromb Haemost 1996;76:135–8. 8865518

[3] Kazama M Tahara C Suzuki Z . Abnormal plasminogen, a case of recurrent thrombosis. Thromb Res 1981;21:517–22. 7268697 10.1016/0049-3848(81)90154-7

[4] Aoki N Moroi M Sakata Y . Abnormal plasminogen. A hereditary molecular abnormality found in a patient with recurrent thrombosis. J Clin Invest 1978;61:1186–95. 659588 10.1172/JCI109034 PMC372639

[5] Sakata Y Aoki N. Molecular abnormality of plasminogen. J Biol Chem 1980;255:5442–7. 6445365

[6] Okamoto A Sakata T Mannami T . Population-based distribution of plasminogen activity and estimated prevalence and relevance to thrombotic diseases of plasminogen deficiency in the Japanese: the Suita Study. J Thromb Haemost 2003;1:2397–403. 14629475 10.1046/j.1538-7836.2003.00419.x

[7] Shinmyozu K Maruyama Y Osame M . Blood coagulation studies in a family with congenital plasminogen abnormality--with a special relevance to the association of thrombotic tendency with abnormal plasminogen. Rinsho Ketsueki 1986;27:133–9. 3723817

[8] Ichinose A Espling ES Takamatsu J . Two types of abnormal genes for plasminogen in families with a predisposition for thrombosis. Proc Natl Acad Sci U S A 1991;88:115–9. 1986355 10.1073/pnas.88.1.115 PMC50760

[9] Demarmels Biasiutti F Sulzer I Stucki B . Is plasminogen deficiency a thrombotic risk factor? A study on 23 thrombophilic patients and their family members. Thromb Haemost 1998;80:167–70. 9684804

[10] Tait RC Walker ID Conkie JA . Isolated familial plasminogen deficiency may not be a risk factor for thrombosis. Thromb Haemost 1996;76:1004–8. 8972025

[11] Tashima Y Banno F Kita T . Plasminogen Tochigi mice exhibit phenotypes similar to wild-type mice under experimental thrombotic conditions. PLoS One 2017;12:e0180981. 28686706 10.1371/journal.pone.0180981 PMC5501636

[12] Ooe A Kida M Yamazaki T . Common mutation of plasminogen detected in three Asian populations by an amplification refractory mutation system and rapid automated capillary electrophoresis. Thromb Haemost 1999;82:1342–6. 10544925

[13] Kim HJ Seo JY Lee KO . Distinct frequencies and mutation spectrums of genetic thrombophilia in Korea in comparison with other Asian countries both in patients with thromboembolism and in the general population. Haematologica 2014;99:561–9. 24162787 10.3324/haematol.2013.092023 PMC3943322

